# Insights Into Immunothrombotic Mechanisms in Acute Stroke due to Vaccine-Induced Immune Thrombotic Thrombocytopenia

**DOI:** 10.3389/fimmu.2022.879157

**Published:** 2022-05-10

**Authors:** Nicole de Buhr, Tristan Baumann, Christopher Werlein, Leonie Fingerhut, Rabea Imker, Marita Meurer, Friedrich Götz, Paul Bronzlik, Mark P. Kühnel, Danny D. Jonigk, Johanna Ernst, Andrei Leotescu, Maria M. Gabriel, Hans Worthmann, Ralf Lichtinghagen, Andreas Tiede, Maren von Köckritz-Blickwede, Christine S. Falk, Karin Weissenborn, Ramona Schuppner, Gerrit M. Grosse

**Affiliations:** ^1^ Department of Biochemistry, University of Veterinary Medicine Hannover, Hannover, Germany; ^2^ Research Center for Emerging Infections and Zoonoses (RIZ), University of Veterinary Medicine Hannover, Hannover, Germany; ^3^ Department of Neurology, Hannover Medical School, Hannover, Germany; ^4^ Institute of Pathology, Hannover Medical School, Hannover, Germany; ^5^ Clinic for Horses, University of Veterinary Medicine Hannover, Hannover, Germany; ^6^ Institute of Diagnostic and Interventional Neuroradiology, Hannover Medical School, Hannover, Germany; ^7^ Member of the German Center for Lung Research (DZL), Biomedical Research in Endstage and Obstructive Lung Disease Hannover (BREATH), Hannover, Germany; ^8^ Institute of Clinical Chemistry, Hannover Medical School, Hannover, Germany; ^9^ Department of Hematology, Hemostasis, Oncology and Stem Cell Transplantation, Hannover Medical School, Hannover, Germany; ^10^ Institute of Transplant Immunology, Hannover Medical School, Hannover, Germany

**Keywords:** immunothrombosis, neutrophil extracellular traps (NETs), stroke, complement, cytokines, vaccination

## Abstract

During the COVID-19 pandemic, vaccination is the most important countermeasure. Pharmacovigilance concerns however emerged with very rare, but potentially disastrous thrombotic complications following vaccination with ChAdOx1. Platelet factor-4 antibody mediated vaccine-induced immune thrombotic thrombocytopenia (VITT) was described as an underlying mechanism of these thrombotic events. Recent work moreover suggests that mechanisms of immunothrombosis including neutrophil extracellular trap (NET) formation might be critical for thrombogenesis during VITT. In this study, we investigated blood and thrombus specimens of a female patient who suffered severe stroke due to VITT after vaccination with ChAdOx1 in comparison to 13 control stroke patients with similar clinical characteristics. We analyzed cerebral thrombi using histological examination, staining of complement factors, NET-markers, DNase and LL-37. In blood samples at the hyper-acute phase of stroke and 7 days later, we determined cell-free DNA, myeloperoxidase-histone complexes, DNase activity, myeloperoxidase activity, LL-37 and inflammatory cytokines. NET markers were identified in thrombi of all patients. Interestingly, the thrombus of the VITT-patient exclusively revealed complement factors and high amounts of DNase and LL-37. High DNase activity was also measured in blood, implying a disturbed NET-regulation. Furthermore, serum of the VITT-patient inhibited reactive oxygen species-dependent NET-release by phorbol-myristate-acetate to a lesser degree compared to controls, indicating either less efficient NET-inhibition or enhanced NET-induction in the blood of the VITT-patient. Additionally, the changes in specific cytokines over time were emphasized in the VITT-patient as well. In conclusion, insufficient resolution of NETs, e.g. by endogenous DNases or protection of NETs against degradation by embedded factors like the antimicrobial peptide LL-37 might thus be an important factor in the pathology of VITT besides increased NET-formation. On the basis of these findings, we discuss the potential implications of the mechanisms of disturbed NETs-degradation for diagnostic and therapeutic approaches in VITT-related thrombogenesis, other auto-immune disorders and beyond.

## Introduction

Vaccination is one of the most important countermeasures in controlling the coronavirus disease 2019 (COVID-19) pandemic. The ChAdOx1 vaccine (AZD1222) was tested to be efficacious against COVID-19 and demonstrated a favorable safety profile, according to an interim analysis of randomized controlled trials comprising more than 11,000 participants (1). After approval and application of the ChAdOx1 vaccine in the general population, however, pharmacovigilance concerns emerged with a very rare but potentially disastrous complication, i.e. cerebral venous sinus thrombosis (CVST), leading to temporal suspension of this vaccine in several countries. Since spring 2021, numerous cases of CVST have been reported after vaccination (2), each of them being accompanied by thrombocytopenia. The potential mechanism behind this pro-thrombotic state was described as vaccine induced immune thrombotic thrombocytopenia (VITT) that is suspected to be mediated by antibodies against platelet factor 4 (PF4) (1–3). The thrombotic manifestations of VITT are not limited to the cerebral venous system but occur also in other venous and arterial territories, including the brain supplying vessels and are therefore a rare cause of ischemic stroke (4–6).

Greinacher *et al.* reported that the interplay of PF4, antibodies and activated platelets might contribute to the formation of neutrophil extracellular traps (NETs) which are known to be crucial pro-thrombotic effectors in immunothrombosis (7). In a recent observational study of healthy individuals being vaccinated with ChAdOx1 (n=138) or BNT162b2 (n=143), Thiele *et al.* reported a post-vaccination anti-PF4 antibody prevalence of 6.8% (95%CI, 4.4% - 10.3%) (8). However, none of the anti-PF4 antibody positive samples were shown to be platelet activating in the presence of PF4 (8).

The exact mechanisms behind VITT and its thrombotic complications are thus still insufficiently understood. Most importantly, predictors of adverse events due to VITT as well as targeted therapeutic approaches are lacking. In this study, we aimed to provide new insights into potential mechanisms of immunothrombosis in VITT. We analyzed harvested thrombus and blood samples of a previously healthy woman who suffered a severe ischemic stroke ten days after application of the ChAdOx1 vaccine, as well as of 13 patients suffering from acute ischemic stroke without signs of VITT as a control group. In all patient samples we conducted histological examination and quantified NET-markers, DNase activity, the antimicrobial peptide LL-37 and distinct cytokines in blood at onset and 7 days after stroke onset.

## Materials and Methods

### Patient Collective

We report on analyses of blood and thrombus tissue of a female patient (index patient) who suffered severe ischemic stroke due to a thrombotic L-occlusion of the right internal carotid and the middle cerebral artery (MCA) that occurred on March 26th 2021 and that was successfully treated with mechanical thrombectomy (MT) at Hannover Medical School, Germany. As control group, we considered 13 patients with acute ischemic stroke due to proximal large vessel occlusion (LVO) in the anterior circulation who also underwent MT and revealed comparable clinical characteristics. For all patients, demographic and clinical data were obtained. Arterial blood specimens were collected immediately before MT and peripheral venous blood was drawn at a follow-up one week later. In each case, the thrombus retrieved during MT was histologically analyzed, as described below. Stroke severity was graded using the National Institutes of Health Stroke Scale (NIHSS) at admission. Reperfusion status was reported using the modified Treatment in Cerebral Infarction (mTICI) score. Vascular risk factors were subsumed with the Essen Stroke Risk Score (ESRS).

### Blood Sample Collection

We collected arterial blood samples in all patients immediately before MT and in addition peripheral venous blood at day 7 (7d). Plasma and serum samples were stored at -80°C until the respective assays were performed.

### Measurement of NET Markers (cfDNA, Histone-Myeloperoxidase (MPO)

We analyzed all samples with commercially available ELISA or in-house established ELISA. A Quant-iT ™ PicoGreen^®^ assay (Invitrogen, Carlsbad, California, USA, P11496) was used as described previously (9, 10), to determine the amount of free DNA in the plasma samples. The amount of cell-free DNA (cfDNA) in each sample was calculated based on the standard curve.

A sandwich ELISA for the detection of histone-MPO complexes in serum was established. The streptavidin-coated microplate of the Cell Death Detection ELISAPLUS Kit (Roche 11774425001) was coated with 100 µL per well of an anti-histone-biotin antibody (component 1 of the same ELISA Kit) for two hours at room temperature on a microplate shaker at 200 rpm. The antibody was beforehand diluted 1:20 in 1x phosphate-buffered saline (PBS, Sigma-Aldrich P5493-1l, 10× PBS diluted to 1× PBS in distilled water). Then, the wells were washed twice with 100 µL incubation buffer (component 4 of the ELISA Kit) per well and blocked with 100 µL incubation buffer per well for one hour at room temperature while shaking at 200 rpm. 100 µL serum/well was incubated for 1.5 hours at room temperature on a microplate shaker at 300 rpm. Buffer was included as blank, as well as an inhouse-made standard as positive control. This standard is composed of 1.25 µg DNA, isolated from human neutrophils, and 1.25 µg MPO (Recombinant Human Myeloperoxidase Protein, R&D systems 3174-MP), incubated together for 30 minutes at 37°C, 5% CO_2_. This solution was then filled up to a total volume of 100 µL with incubation buffer. After incubation of the samples, the wells were washed three times with 0.05% PBS-Tween20 (Roth 9127.2), followed by incubation with 100 µL rabbit anti-human MPO antibody [Merck Millipore #07-496-I, 1 mg/mL; 1:200 diluted in 1% PBS-BSA (bovine serum albumin, Roth CP84.2 or 1ETA.2)] for 1.5 hours at room temperature and 250 rpm. After another three washing steps with 0.05% PBS-Tween20, an incubation with goat anti-rabbit IgG HRP conjugate (Merck Millipore #12-348, 1:5000 diluted in PBS) for one hour at room temperature at 250 rpm was performed. 100 µL of TMB ELISA Substrate (High Sensitivity, abcam ab171523) were added for 25 minutes after three washes. The reaction was stopped by 100 µL 450 nm Stop Solution for TMB Substrate (abcam ab171529). Readouts were performed by a plate reader (Multiscan Go, Thermo Scientific N13133) at 450 nm minus blank value.

### Measurement of LL-37 in EDTA Plasma

LL-37 ELISA Kit (HycultBiotech, Uden, The Netherlands, HK321-02) was used to determine the amount of LL-37 in EDTA-plasma. The test was performed following the manufacturer’s instructions with 1:20 diluted samples.

### Measurement of DNase Activity and Myeloperoxidase (MPO) Activity in Serum

DNase I Activity Assay Kit (BioVision, Milpitas, California, USA, Fluorometric, K429-100) was used to determine the DNase I activity in serum. The test was performed following the manufacturer’s instructions with 25 µL for each sample.

Myeloperoxidase Colorimetric Activity Assay Kit (Sigma Aldrich, St. Louis, Missouri, USA, MAK068) was used to determine the MPO activity in serum. The test was performed following the manufacturer’s instructions with 10 µL for each sample and a respective blank for each individual sample. The assay was performed for 30 minutes and based on all variables the enzyme activity was calculated.

### Fibrinogen in Citrate-Plasma

A fibrinogen detection kit (DiaSys, Flacht, Germany, 30300) was used to determine fibrinogen in citrate plasma. The assay was conducted following the manufacturer’s instructions with 1:10 diluted samples using a Merlin coagulometer 4 Plus (Merlin medical, Lemgo, Germany).

### Measurements of Cytokines and Growth Factors in Plasma

Concentrations of cytokines and growth factors were measured using the Luminex-based MILLIPLEX MAP Human Cytokine/Chemokine Panel (HCYTA-60K-PX38, Merck Millipore, Darmstadt, Germany) in EDTA-Plasma according to the manufacturer’s instructions. Following analytes were determined: epidermal growth factor (EGF), interleukin (IL)-1β, Eotaxin (CCL11), granulocyte colony-stimulating factor (G-CSF), granulocyte macrophage colony-stimulating factor (GM-CSF), fractalkine (CX_3_CL1), interferon (IFN)-α2, IFN-γ, IL-10, IL-12p40, IL-12p70, IL-13, IL-15, IL-17A, IL-1RA, IL-1α, IL-2, IL-3, IL-4, IL-5, IL-6, IL-7, CXCL8/IL-8, IFN-γ induced protein 10 (IP-10/CXCL10), monocyte chemotactic protein 1 (MCP-1/CCL2), macrophage inflammatory protein 1 α (MIP-1α, CCL3), macrophage inflammatory protein 1 β (MIP-1β/CCL4), tumor necrosis factor (TNF)-α, TNF-β, vascular endothelial growth factor (VEGF)-A, IL-17E/IL-25, IL-17F, IL-18, IL-22, macrophage colony-stimulating factor (M-CSF), monokine induced by IFN-γ (MIG)/CXCL9, and platelet derived growth factor (PDGF)-AA, PDGF-AB/BB.

### Isolation Human Neutrophils

Human granulocytes were isolated from fresh whole heparin blood of healthy donors using the Polymorph Prep system (Progen; 1.113 g/mL, 1114683) as previously described (9). The cells were stained with trypan blue (1680.1 Carl Roth^®^, Karlsruhe, Germany) and counted in Neubauer chamber to adjust for a specific cell number.

### NET Induction Assay With Serum

The NET induction assay was performed with human neutrophils as described previously (11) with slight changes. Summarized briefly, cover slips (8 mm; Thermo Fisher Scientific (Bremen) GmbH) were placed into a 48 well plate and coated with poly-L-lysine. In each well 2x10^5^ neutrophils/100 µL were seeded. The total volume with stimulus was 200 µL. As a negative control, RPMI 1640 medium (ThermoFisher, 11835063, Waltham, MA, USA) was added. As a positive control, phorbol 12-myristate 13-acetate (PMA, 25 nM final concentration, 524400 Sigma Aldrich, Munich, Germany) was added. The neutrophils were stimulated with 25 µL serum with and without PMA. The plate was incubated at 37°C, 5% CO_2_. After 1 h and 3 h of incubation the plate was centrifuged (250 g, 5 min). Samples were fixed with paraformaldehyde (4% final concentration, Science Services, E15710-250, Munich, Germany) and the plate were wrapped with parafilm and stored at 4°C until the staining was conducted.

### NET Induction Assay With PF4 and AntiPF4

A 96-well plate with glass bottom (MatTek Corporation, P96G-1.5-5-F, Ashland, MA, USA) was used. Each well was coated in accordance with the manufacturer’s instructions with poly-L-lysine (0.01% solution P4707, Sigma Aldrich, Munich, Germany) and handled afterwards as previously described (9). In each well 1x10^5^ neutrophils/50 µL were seeded. The total volume with stimulus was 100 µL. As a negative control, RPMI 1640 medium was added. As a positive control, 50 µL PMA (25 nM final concentration) was added. The neutrophils were stimulated with PF4 (SRP3142-20UG Sigma Aldrich PF-4 (CXCL4) human, reconstituted in water (Sigma Aldrich, W4502), final concentration 2µM as described previously (12)) or antiPF4 (IgG1 mouse anti human PF-4 (D-7): sc-398979, Santa Cruz Biotechnology, INC; each vial contains 200µg in 1 mL PBS, Stock 200µg/mL, final concentration 1µg/100µL as described previously (12)) and a combination of both. As internal control in one run neutrophils were stimulated with water (Sigma Aldrich, W4502) only. The amount was the same as used for PF4 stimulation. Furthermore, neutrophils were treated with a respective isotype control (IgG1 from murine myeloma (0.2 mg/mL), with the same concentration as the antiPF4. The plate was centrifuged (250 g, 5 min) and was incubated at 37°C, 5% CO_2_. After 2 h of incubation the plate was centrifuged again (250 g, 5 min). Samples were fixed as described above and the plate was wrapped with parafilm and stored at 4°C until the staining was conducted.

### Histological Thrombus Analysis

Thrombi were retrieved during mechanical thrombectomy and fixed in 4% buffered formalin immediately after removal. Specimens were embedded in paraffin. 2 µm thick sections were cut followed by histological staining using Hematoxylin and Eosin (HE), Elastica van Gieson (EvG) and periodic acidic shift reaction (PAS) at the Institute of Pathology at Hannover Medical School. Immunohistochemistry for C1q Dilution 1:16000 (A0136, Agilent Dako, California, USA), C3d Dilution 1:100 (403A-76, Agilent Dako, California, USA), C4d RTU (760-4803, Hoffmann-La Roche, Basel, Swiss), IgA Dilution 1:40000 (A0262, Agilent Dako, California, USA), IgG Dilution 1:60000 (A0423, Agilent Dako, California, USA), IgM Dilution 1:10000 (A0425 Agilent Dako, California, USA) and vWF Dilution 1:40 (M0616, Agilent Dako, California, USA) were stained on a VENTANA BenchMark ULTRA (Hoffmann-La Roche, Basel, Switzerland) with the aforementioned dilutions and pretreatment and incubation times according to the manufacturers advice. The histological analysis was performed blinded to clinical data on a routine diagnostic light microscope (BX43, Olympus, Tokyo, Japan). Representative images were acquired with an Olympus CS50 camera (Olympus, Tokyo, Japan) using Olympus cellSens Software (Olympus, Tokyo, Japan) on the above mentioned routine diagnostic light microscope. Image processing was carried out in ImageJ software and “Fiji: an open-source platform for biological-image analysis” (13).

### NET Staining and Analysis

NETs were stained after the permeabilization and the blocking of the samples as previously described (11). Summarized briefly, the following antibodies were used: a mouse monoclonal antibody (IgG2a) against DNA/histone 1 (MAB3864; Sigma Aldrich, Millipore 0.55 mg/mL diluted 1:1000, Billerica, MA, USA), a rabbit antibody (IgG) against anti-human myeloperoxidase (A039829-2 Agilent, Santa Clara, CA, USA, 3.3 mg, 1:300), a rabbit antibody (IgG) against anti-human H3cit (abcam ab5103, 1:31) or a mouse monoclonal antibody (IgG1) against LL-37/CAP-18 (HM2070, 100 µg/mL diluted 1:25), for 1 h at room temperature.

For the isotype controls murine IgG2a (from murine myeloma, M5409-1mg conc. 0.2 mg/mL, 1:364 Sigma Aldrich, Munich, Germany), rabbit IgG (from rabbit serum, Sigma Aldrich, Munich, Germany, I5006, 1.16 mg/mL, 1:105 or 1:36) and IgG1 (from murine myelom, M5284-1mg conc. 0.2 mg/mL, Sigma Aldrich, Munich, Germany, 1:50) were used. As secondary antibodies, goat anti-mouse Alexa 488Plus (1:500, Invitrogen, Carlsbad, CA, USA) and goat anti-rabbit Alexa 633 (Invitrogen, Carlsbad, CA, USA, 2 mg, 1:500, Waltham, MA, USA) were used and incubated for 1 h at room temperature in the dark. In the 48 well plate the samples were stained 10 min (in the dark, room temperature) with aqueous Hoechst 33342 (1:1000, stock 50 mg/mL, Sigma Aldrich, Munich, Germany) and finally embedded in ProlongGold (Invitrogen, P36930). In the 96 well plate samples were washed inside the plate and instead of the Hoechst staining embedded in 20 µL ProlongGold with DAPI (Invitrogen, P36931). Samples were stored at 4°C in the dark until analysis.

### NET Quantification

The respective isotype control (PMA stimulated) was used for the microscope settings. The six pictures per sample were taken randomly by Leica TCS SP5 AOBS confocal inverted-base fluorescence microscope with an HCX PL APO ×40 0.75–1.25 oil immersion objective and the total number of NET-releasing neutrophils were counted as described previously (11). In the NET induction assay with PF4 and antiPF4 the area covered with NETs were quantified in six randomly imaged areas as described previously (14). The analysis was conducted using ImageJ 1.53J.

### DNA-Histone-1 Complexes/LL-37/Myeloperoxidase/DNase Examination in Paraffin Sections

For DNA-Histone-1 complexes**/**LL-37/myeloperoxidase (MPO) and DNase detection, serial cuts of paraffin sections of thrombi were analyzed. The immunofluorescence staining of paraffin section was performed as previously described (15) with the following first antibodies, respectively: 1. mouse IgG2a anti-DNA/histone-1 complexes (Millipore MAB3864, Sigma Aldrich, 0.55 mg; 1:100), 2. mouse IgG1 anti-LL-37 CAP-18 (HM2070, 100 µg/mL diluted 1:25), 3.rabbit anti-human myeloperoxidase antibodies (Dako A0398, 3.3 mg, 1:300) and 4. rabbit anti-DNase 1 (Invitrogen; PA5-22017, Carlsbad, CA, USA; 1:100). All were dissolved in blocking buffer and incubated overnight at 4°C. As secondary antibodies, a goat anti-mouse antibody (Alexa 488PLUS, Invitrogen) and goat-anti rabbit antibody (Alexa 568, Invitrogen) were used diluted 1:500 in blocking buffer. Respective isotype controls were included. At the end, all samples were processed with TrueVIEW autofluorescence quenching kit (Vector laboratories) following the manufacturer’s instructions and counter stained with Hoechst 33342 (1:1000, stock 50 mg/mL, Sigma Aldrich, Munich, Germany). Samples were recorded using a Leica TCS SP5 AOBS confocal inverted-base fluorescence microscope with an HCX PL APO ×40 0.75–1.25 oil immersion objective or HCX PL APO 63 × 1.40 oil objective. The settings were adjusted using isotype control antibodies in separate preparations. Stitched images were combined using Adobe Photoshop CS6.

The raw integrated density was measured using ImageJ 1.53J for the green and red channel separately in randomly taken images (115: n=3, 259: n=9, 325: n=8 and 357: n=8 images). The analyzed samples were stained and imaged at the same time.

To substantiate a potential overlap of selected markers that could represent potential functional subsets of LL-37 and DNase, the EzColocalization plugin for ImageJ (version 1.53c; http://imagej.nih.gov.ij/) was exemplarily applied to selected images captured with a confocal microscope (16). Single images that were used to measure the integrated density and single images from one z-stack of the VITT-patient were investigated using Spearman’s rank correlation coefficient (SRCC) for multicolor image correlation *via* ranking of pixel intensity values (-1= perfect negative association of ranks; 0 = no association; 1 = perfect positive association of ranks). The SRCC does not evaluate the degree of overlap between both channels but predicts signal intensities within the same pixel, which can be used as an indicator of potential functional correlation (17).

### DNase Digestion of Paraffin Sections

The digestion of NETs in paraffin section with external DNase was conducted as described previously (15). Briefly, the digestion was conducted in one slide for 30 minutes at 37°C. A serial cut of the paraffin section was handled and stained in parallel without adding DNase. Samples were recorded using a Leica TCS SP5 AOBS confocal inverted-base fluorescence microscope with an HCX PL APO ×40 0.75–1.25 oil immersion objective The settings were adjusted using isotype control antibodies in separate preparations. Stitched images were combined using Adobe Photoshop CS6.

### Ethical Study Approvals

We collected blood samples for neutrophil isolation from healthy volunteers after written informed consent. This was approved by the Ethics Committee of Hannover Medical School, Hannover, Germany, and registered under no. 3295–2016.

Blood and thrombus samples were also collected in accordance with the approval of the Ethics Committee at Hannover Medical School (vote no. 7689). All patients or proxies provided written informed consent.

### Statistical Analysis

Data were analyzed using Excel 2021 (Microsoft) and GraphPad Prism, version 9.0.121.0. Normal distribution was tested using the Shapiro Wilk normality test. Differences and relations between groups were investigated and interpreted as described in the figure legends.

## Results

### Clinical Characteristics

The index patient is a 61-year-old female who was in her usual state of health with an obese state and hypothyroidism requiring substitution therapy. She has been vaccinated against SARS-CoV-2 using the ChAdOx1 vaccine on March 26^th^ 2021. On April 4^th^ 2021 she woke up with headache but without any neurological symptoms. A few hours later, she awoke with a left sided hemiparesis. She was referred to a primary care clinic and was diagnosed of having an ischemic stroke in the right MCA territory (NIHSS : 17) due to thrombotic occlusion of the right carotid artery from the carotid bulb to the MCA. Of note, the computed tomographic angiography revealed no signs of atherosclerotic disease. The patient underwent a recanalization procedure including aspiration and stenting at our center, finally leading to a reperfusion status according to a modified treatment in cerebral infarction score (mTICI) of 2B. A thrombus of approximately 7cm in length was retrieved. Follow-up imaging revealed extensive left hemispheric brain infarction with swelling and hemorrhagic transformation. The patient therefore underwent decompressive craniectomy on the same day.

Cell counts in peripheral blood revealed a thrombocytopenia of 62/nL on admission and 25/nL on day three (reference interval: 150-370/nL). D-dimer was massively elevated to >35.2mg/L (reference interval: <0.5mg/L). Anti PF4 antibodies were detected and the patient was treated with intravenous immunoglobulins (IVIG), dexamethasone and argatroban as described previously (4) with good effect on platelet counts. During the inpatient course, further thrombotic complications were diagnosed including thromboses of an arm vein, superficial cubital veins, the left-sided jugular vein, as well as an arterial occlusion of a popliteal artery, and a pulmonary artery occlusion. The diagnostic work-up revealed no competing stroke etiology besides VITT.

Clinical details of the control group are summarized in **Table 1**. These 13 patients had acute ischemic stroke due to LVO in the anterior circulation and were not vaccinated against SARS-CoV-2, nor suffered COVID-19.

**Table 1:** Clinical characteristics of the study collective.

	Index patient	Control patients
N	1	13
Age (years)	61	Median (Q1-Q3): 67 (61–80)
Sex (female)	1	7 (54%)
Stroke Etiology	ESUS (VITT)	ESUS: 4 (31%) LAA: 5 (38%) CES: 4 (31%)
ESRS	0	Median (Q1-Q3): 3 (2–3)
NIHSS	17	Median (Q1-Q3): 17 (15–18)

CES, cardioembolic stroke; ESRS, Essen Stroke Risk Score; ESUS, embolic stroke of undetermined source; LAA, large artery atherosclerosis; NIHSS, National Institutes of Health Stroke Scale.

### Histological, Immunohistological and Immunofluorescence Analysis of Thrombi Detected Complement Factors and NETs in VITT

**Figure 1:**
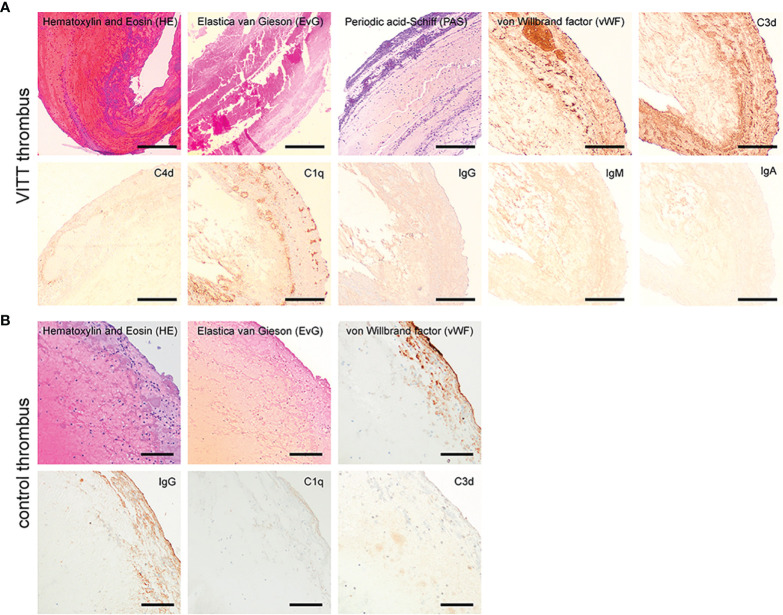
Histopathological examination of the VITT-thrombus and a control. **(A)** On conventional histology the VITT-thrombus displayed a mixed content of erythrocyte rich (red in H&E) and platelet rich areas (purple in PAS-reaction) without signs of organization or parts of vessel walls in a staining for connective tissue (EvG). On further investigation *via* immunohistochemistry (dark brown coloration = positive signal), the thrombus showed areas of increased content of von Willebrand-factor as seen in other thrombi. Further, presence of complement factors C1q and C3d alongside a faint positive staining for IgG-immunoglobulin was present, while IgM, IgA and C4d could not be detected in the thrombus material. Magnification 40x, scale bar 300µm. **(B)** On conventional histology, the control thrombus of a stroke patient from the cohort displayed a mixed content of erythrocyte rich (H&E staining) and platelet rich areas without signs of organization or parts of vessel walls in a staining for connective tissue (EvG) comparable to the VITT-thrombus. On further investigation *via* immunohistochemistry (dark brown coloration = positive signal) the thrombus showed a comparable increase of von-Willebrand-factor and IgG-Immunoglobulin as described for the thrombus in **(A)** However, no presence of complement factors C1q and C3d could be detected. Magnification 100x, scale bar 100µm.

**Figure 2:**
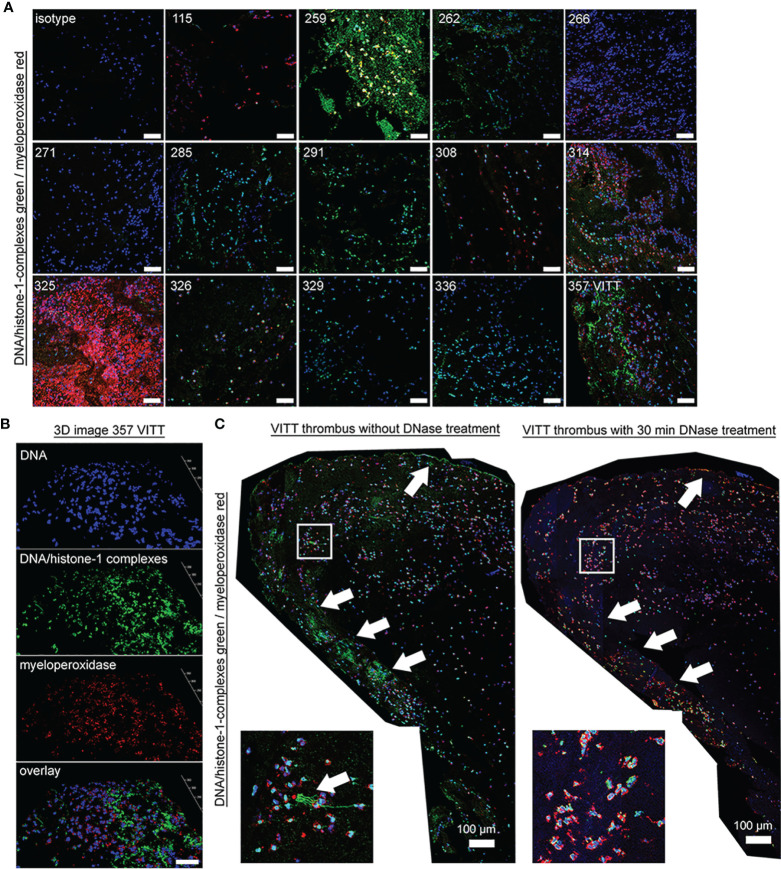
Specific NET markers in thrombi of VITT and controls are detectable. **(A)** NET markers DNA/histone-1-complexes (green) and myeloperoxidase (red) were stained in extracted thrombi of the VITT-patient (357) and all 13 control patients (blue = DNA). Microscope settings were adjusted to the presented isotype control and exemplary pictures are presented. **(B)** 3D image of z-stacks from VITT-thrombus were constructed with LAS X 3D Version 3.1.0 software (Leica), (6.21 µm consisting of 38 sections, scale bar = 50µm.) **(C)** Stitched images show distribution of NETs inside the VITT-thrombus (blue = DNA, green = DNA-histone-1-complex, red = myeloperoxidase). White arrows depict strong NET signals (DNA-histone-1-complex and myeloperoxidase) in the left panel, especially in the outer range. A zoom picture of the white squared area is presented. Specificity of NET markers were identified after DNase degradation. Serial cuts of thrombi were treated during the NET-staining with and without DNase for 30 minutes at 37°C. DNase treatment deletes the DNA-histone-1-complex from thrombus section, but not the myeloperoxidase. The settings were adjusted to a respective isotype control (with and without DNase treatment).

The thrombus of the index case revealed a mixed content of erythrocyte rich and platelet rich areas and the detection of von Willebrand factor (vWF) without signs of organization or parts of vessel walls in a staining for connective tissue. A similar mixture of erythrocyte- and platelet-rich areas with vWF was identified in the control thrombi. Interestingly, complement factors C1q and C3d, and slightly IgG were only detectable inside the VITT-thrombus and not inside all 13 control thrombi upon immunohistochemistry (**Figure 1** and **S1**). Interestingly, complement was especially positive in the outer shell of the thrombus. In accordance to this finding, Pitkänen *et al.* recently proposed that VITT is associated with a strong activation of the complement system leading to aggregation of healthy platelets (18). *Leffler et al.* showed that in patients with Systemic Lupus Erythematosus an insufficient degradation of NETs is associated with deposition of complement factors with a subsequent pro-inflammatory response (19). Interestingly, complement C1q, which we were able to detect exclusively in the thrombus of the VITT-patient, prevents NETs of being degraded by DNase. A bidirectional interrelation between NETs and the complement system has accordingly been postulated in coagulation processes (20). This would be in line with our finding of complement and NET-deposition in the outer shell of the VITT-thrombus (21) (**Figures 1**, **2** and **S1, S2**). Furthermore, the binding of components of the contact system (kininogen and factor XII) to NETs and the activation was shown (22).

**Figure 3:**
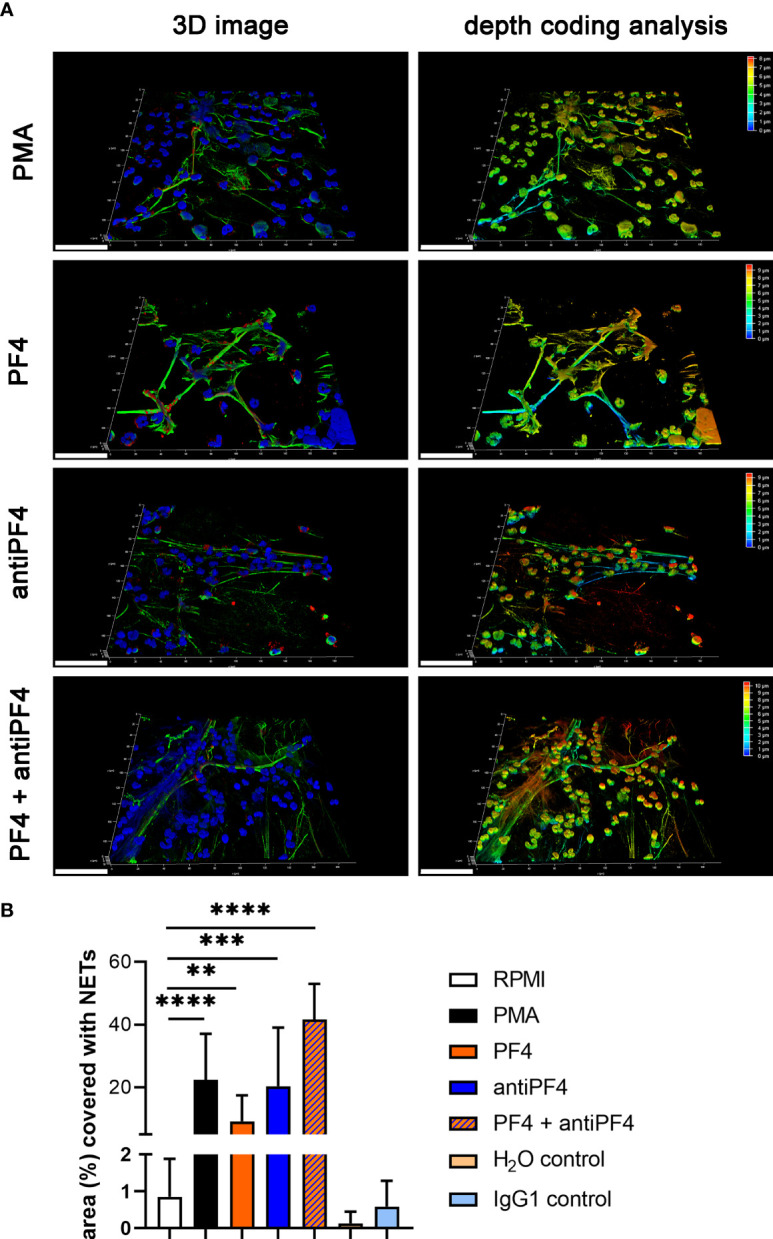
PF4 and antiPF4 are potential NET inducer forming compact NETs. Human blood-derived neutrophils release NETs after 2 h incubation in response to PF4 and antiPF4. NETs were detected by confocal immunofluorescence microscopy. The settings were adjusted to a respective isotype control. **(A)** Representative 3D reconstructions of z-stacks are presented, showing extracellular DNA-fibers positive for DNA/histone-1 complex (green) and myeloperoxidase (red), (Scale bar = 50 µm). Images were constructed with LAS X 3D Version 3.1.0 software (Leica): PMA = 8.09 µm consisting of 49 sections; PF4 = 9.57 µm consisting of 58 sections; antiPF4 = 9.40 µm consisting of 57 sections; PF4 and antiPF4 = 10.41 µm consisting of 63 sections. Out of this 3D volume a depth coding analysis was conducted and is presented with a depth coding scale bar. **(B)** In each experiment and for each sample, six randomly taken pictures from two individual slides were analyzed for NET quantification. The area covered with NETs was measured and used for statistic. In one experiment the internal controls H_2_O and mouse IgG1 were included as control for PF4 (dissolved in H_2_O) and antiPF4 (mouse IgG1 anti human PF4). Data were analyzed with one-tailed unpaired Student’s t-Test calculated always to negative control (RPMI) (**p < 0.01, ***p < 0.001 and ****p < 0.0001). Data are presented with mean ± SD.

Specific NET markers (DNA-histone-1-complexes and myeloperoxidase) were detected by immunofluorescence microscopy inside the thrombus of the VITT-patient and all control patients in different amounts (**Figures 2A, B**). However, no systematic difference in the distribution or quantity of NETs inside the thrombus was observed, neither between the index patient nor among the control group. Nevertheless, DNase treatment confirmed the specificity of the NET staining inside the thrombus of the VITT-patient. After DNase treatment, no DNA-histone-1-complex was positively stained, whereas myeloperoxidase was still detectable (**Figures 2C** and **S2**). In stitched images of the VITT-thrombus a more intense and compact NET signal was detectable in the outer area of the thrombus as described above. Since the patient is showing increased antiPF4 level (4), it might be speculated that antiPF4 was acting as NET-inducer in this patient. We performed an *ex vivo* neutrophil stimulation assay and confirmed that in the absence of platelets, PF4 and antiPF4 induced thick and compact NET-fibers (**Figure 3A**). This goes in line with previous published data describing PF4 and antiPF4 as potential NET-inducers. Indeed, our analysis proof also that the area was most covered with NETs after neutrophils were incubated with a combination of PF4 and antiPF4 whereas the respective controls did not induce NETs (**Figure 3B**). As the quantification of NET markers inside histological samples can be influenced by numerous factors, we additionally conducted NET-marker quantification inside the blood.

### NET-Marker Quantification in Blood Identified Increased NET-Formation Over Time and Highest DNase Activity Following VITT-Related Stroke

**Figure 4:**
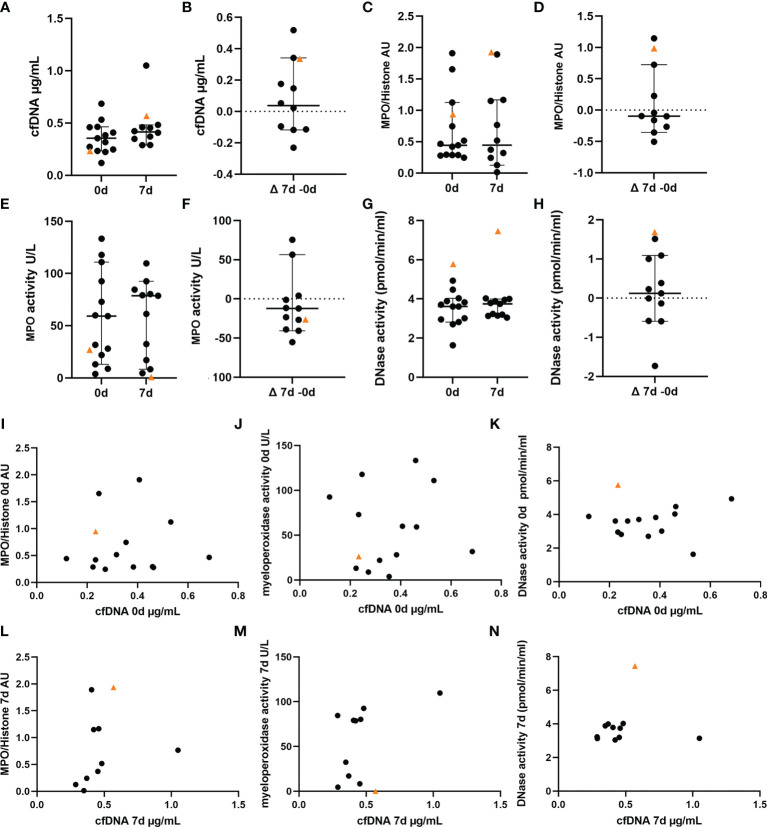
NET markers in blood of VITT and control patients show highest DNase activity in VITT-patient. A-K NET marker analysis in blood of VITT-patient (orange triangle) and control patients (black dots) were measured in blood at onset (n = 13) and 7d post thrombectomy (n = 11) **(A, C, E, G)**. The delta of both measurements was calculated **(B, D, F, H)**. **(A, B)** Determination of cell-free DNA (cfDNA), **(C, D)** Determination of myeloperoxidase (MPO)-histone complexes, **(E, F)** Determination of MPO activity, **(G, H)**, Determination of endogenous DNase activity (regulator of NETs). All data are presented as scatter dot plot, black lines depict the median and whiskers depict 95% confidence intervals of control values in **(A–H)**. **(I–N)** Values of cfDNA in comparison with respective NET markers at onset at 7d of disease were plotted in separate graphs.

As NETs consist of a DNA backbone, the amount of extracellular cfDNA was evaluated as a surrogate parameter for NET-formation in peripheral blood. Whereas the VITT-patient showed relatively low cfDNA at onset, an increase was identified at 7d (**Figures 4A, B** **)**. As second more specific NET-marker besides cfDNA (23), histone-MPO complexes, were analyzed and in the VITT-patient showed a value higher than most controls and the highest increase from onset until 7d (**Figures 4C, D**), indicating again the presence of more NETs at 7d. MPO is mainly stored and released from neutrophil granules by viable activated neutrophils and also involved in NETs-formation by contributing to NET decondensation of the nucleus at early stage of NETosis before the neutrophil disintegrates and dies (24, 25). Interestingly, we identified a low MPO activity and a decrease until 7d in the VITT-patient (lowest value compared to all control patients), which indicates late stage of NETosis, a stage where MPO activity is lost (**Figures 4E, F**). Another enzyme that is highly important to balance NET-formation is DNase. DNase causes degradation of NETs for their elimination and therefore reduces detrimental effects by NETs (26). The highest DNase activity at onset and 7d and furthermore the highest increase until 7d was measured in the VITT-patient, indicating a regulatory reaction of the patient on massive NET-formation (**Figures 3G, H**). It could therefore be speculated that this high DNase activity may partially explain the relatively low cfDNA at onset of the disease. Scatter plot analysis (**Figures 4I–K**) revealed a clear phenotype of high cfDNA level despite highest nuclease activity in case of the VITT-patient at onset. Whereas at onset the scatter plots showed with all analyzed samples a distributed pattern, a change in this pattern was observed at 7d (**Figures 4L–N**). Of note, the VITT patient was more separated when studying the relation between MPO/histone and cfDNA (**Figure 4L**) and DNase activity and cfDNA (**Figure 4N**). Together with the low MPO activity, this may be a hint that NET-formation is taking place in the VITT patient even after the stroke event, but with less frequency in the control group. The question arose, why the VITT-patient suffered from thrombosis in association with high NET amount, although a high DNase activity was detected that is known to degrade NETs (27).

### High Levels of LL-37 in Blood at Day 7 and LL-37 and DNase in VITT-Thrombus Indicate High NET-Induction Over Time and Insufficient NET Resolution in VITT-Patient

**Figure 5:**
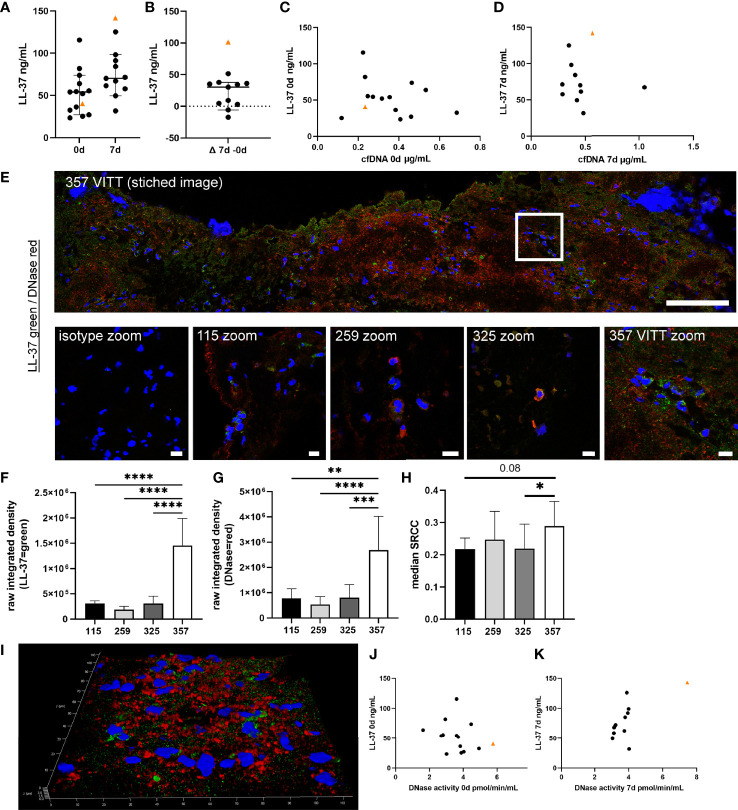
Cationic antimicrobial peptide LL-37, as potential protector of NETs against DNase degradation, is highly increased in VITT-samples. **(A, B)** LL-37, known to induce as well as bind to NETs and stabilize NETs against DNase degradation, was analyzed in EDTA plasma. All data are presented as scatter plots, black lines depict the median and whiskers depict 95% confidence intervals of control values. The VITT-patient is marked as orange triangle. The highest increase of LL-37 over time is detected in the VITT-patient **(B)**. **(C)** Values of cfDNA and LL-37 at onset were plotted in one graph. **(D)** Values of cfDNA and LL-37 at 7d were plotted in one graph. **(E)** LL-37 (green) and DNase (red) were stained in extracted thrombi of the VITT-patient (357) and three control patients without VITT (115, 259 and 325). Stitched image shows distribution of both markers inside the thrombus of the VITT-patient (scale bar = 100µm). A zoom picture of the white squared area is presented. Microscope settings were adjusted to the presented isotype control and example pictures are presented, scale bar = 10µm. **(F, G)** The raw integrated density of LL-37 and DNase was measured in randomly selected pictures. Strong LL-37 and DNase signals in the same area were especially identified in the thrombus of the VITT-patient. All data are presented as mean ± SD. Statistical differences were analyzed by one-way ANOVA, followed by Tukey’s multiple comparisons test (**p < 0.01, ***p < 0.001 and ****p < 0.0001) **(H)** All single images analyzed in **(F, G)** were investigated for colocalization by calculating SRCC. Statistical differences were analyzed by unpaired on-tailed Student’s t-test (*p < 0.05) **(I)** 3D image of z-stacks (42 sections, 0.13µm steps, total size 5.16µm) from VITT-thrombus was constructed with LAS X 3D Version 3.1.0 software (Leica), scale bar = 10µm. In this area high amounts of obviously extracellular DNase and LL-37 were detected. **(J)** Values of DNase activity and LL-37 at onset were plotted in one graph. **(D)** Values of DNase activity and LL-37 at 7d were plotted in one graph.

The cationic antimicrobial peptide LL-37 induces and binds to NETs and protects NETs against DNase digestion (28, 29). In blood samples, LL-37 increased until 7d in six out of 11 control patients (**Figures 5A, B**). The LL-37 level of the VITT-patient was below the median but the increase exceeded those of controls until 7d. Only one control patient showed lower cfDNA/LL-37 values than the VITT-patient at onset (**Figure 5C**) and at 7d the VITT-patient showed the highest cfDNA/LL-37 values (**Figure 5D**), indicating another progress in the VITT-patient. However, free LL-37 unbound to NETs does not protect NETs against DNases and it is uncertain whether unbound LL-37 levels represent an adequate measure of its NET-protective properties. Therefore, we investigated LL-37 inside the thrombus. Indeed, a strong LL-37 signal was observed in the outer area of the VITT-thrombus (**Figure 5E**), whereas semi-quantitatively less LL-37 was detected in three control thrombi (**Figure 5F**). Inside the VITT-thrombus DNase was stained positive in the same area as LL-37 and was semi-quantitatively more detectable (**Figure 5G**). Furthermore, colocalization analysis using the SRCC indicated only a weak colocalization of LL-37 and DNase inside the thrombi of VITT-patient and three control thrombi (**Figure 5H**), as well as in a z-stack out of 42 images (SRCC = 0.30) in the index patient.

Based on the number of nuclei in the investigated area, most LL-37 and DNase was located extracellularly in the VITT-patient, whereas in the controls the markers were identified more cell-associated (**Figures 5E, I**). Interestingly, comparing scatter plots of LL-37/DNase activity of onset and 7d, a strong separation of the VITT-patient was observed, visible especially at 7d (**Figures 5J, K**). In summary, we identified with LL-37 one peptide that potentially might explain a higher NET-induction and impaired DNase degradation of NETs in the VITT-thrombus.

### Comparison of NET-Induction of Activated Neutrophils in Presence of Patient Serum Revealed Highest NET-Formation With Serum From the VITT-Patient

**Figure 6:**
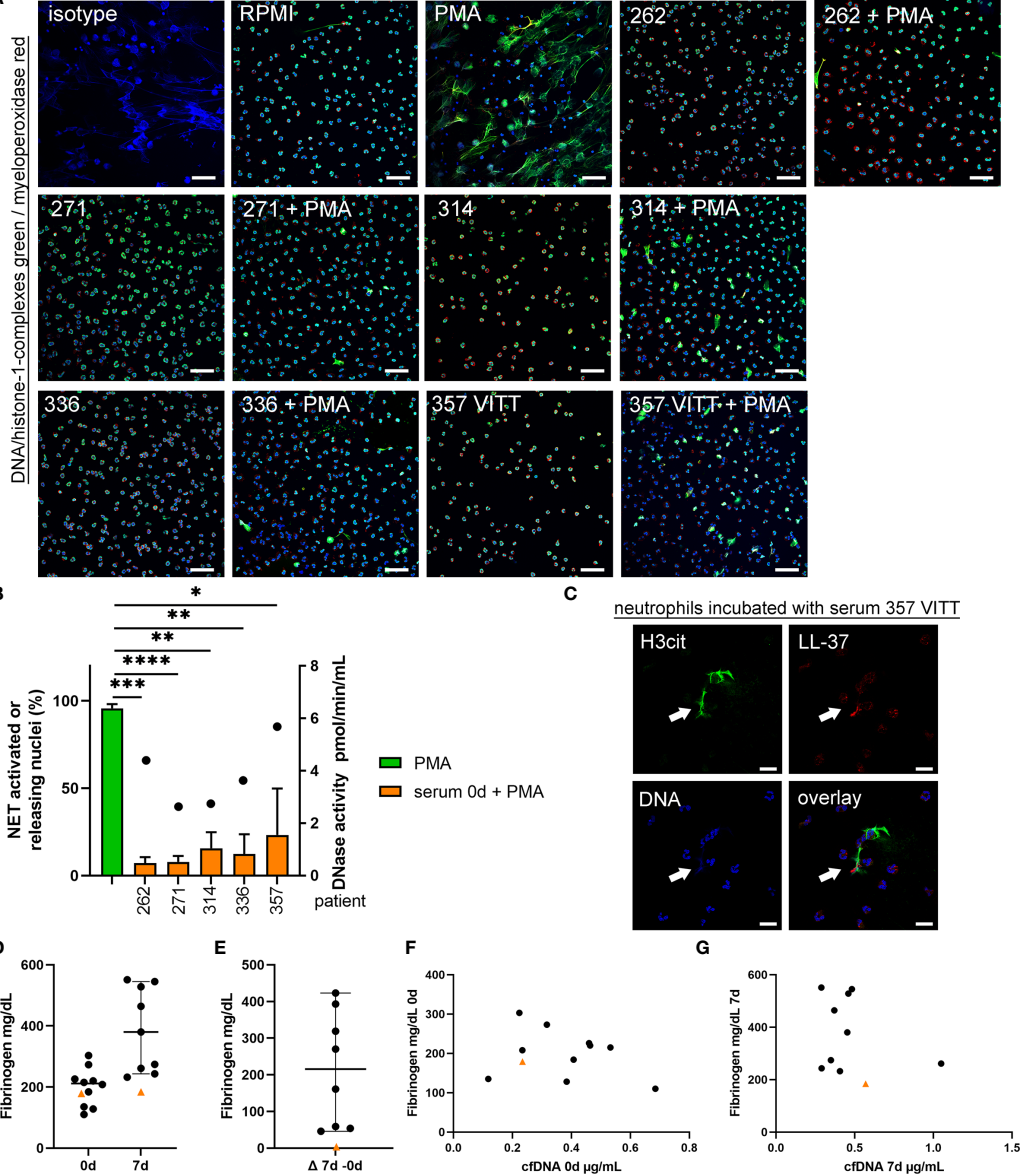
Serum from VITT and control patients does not induce NET-formation, but NET-release from activated neutrophils is less inhibited in VITT-patient. **(A)** Human blood-derived neutrophils were incubated with serum from onset of four control patients and the VITT-patient in presence and absence of the ROS-dependent NET inducer PMA. Samples were analyzed for NETs (blue = DNA, green = DNA-histone-1-complex, red = myeloperoxidase) by confocal immunofluorescence microscopy. The settings were adjusted to a respective isotype control. Representative images (overlay) of all samples incubated with serum, a negative control (RPMI) and a positive control (PMA) are presented. Scale bar = 50µm. **(B)** In each experiment (n = 3) and for each sample, six randomly taken pictures from two individual slides were analyzed for NET quantification. All cells on the six pictures were counted and the mean of activated cells per experiment was calculated and used for statistic. Serum samples without PMA stimulation did not induce NETs (See **Supplementary data Figure S3**). In all serum samples the ROS-dependent NET-induction was significantly reduced. Highest NET-release was detected in the VITT-patient. Data were analyzed with one-tailed paired Student’s t-Test calculated always to PMA positive control (presented with mean ± SD (*p ≤ 0.05, **p < 0.01, ***p < 0.001 and ****p < 0.0001). The DNase activity in the analyzed serum samples, as potential NET degrading enzyme, is presented as dot referring to the right y-axis. **(C)** A representative image is presented, showing an extracellular DNA-fiber positive for citrullinated histone (H3cit) (green) and LL-37 (red) after incubation of neutrophils in serum from the VITT-patient (Scale bar = 20 µm). **(D, E)** Fibrinogen is described to inhibit NET release and was analyzed in citrate plasma. All data are presented as scatter plots, black lines depict the median and whiskers depict 95% confidence intervals of control values (n = 10). Each dot represents one patient. The VITT-patient is marked as orange triangle. A low value is detected in the VITT-patient, without almost no change (delta) over time **(E)**. **(F)** Values of cfDNA and fibrinogen at onset were plotted in one graph. **(G)** Values of cfDNA and fibrinogen at 7d were plotted in one graph.

To quantify NET-formation of PMA activated neutrophils, *ex vivo* assays were performed with neutrophils from healthy donors. NET-formation was quantified in presence of serum. Since serum is known to diminish NETs (30), NET-induction is reduced compared to neutrophils stimulated with PMA without serum (**Figure 6A** and **S3**). Importantly, the activated neutrophils incubated in presence of serum from the VITT-patient showed the highest remaining NET-formation, despite the fact that this patient contains the highest DNase activity (**Figure 6B**). The NET-induction started between one to three hours of incubation, as after one hour no NET-induction was observed (**Figure S3A**), but NETs were detected after three hours (**Figure S3B**). NETs that are induced by serum of the VITT-patient were positive for citrullinated histones 3 and LL-37 and show therefore typical NET markers (**Figure 6C**).

If neutrophils are not activated by PMA, as ROS-stimulating agent, none of the sera induced NET-formation. It might be speculated that the VITT-patient serum contains NET-inhibitors at varying level that impact the results of NET-induction by activated neutrophils. We quantified fibrinogen levels as it may act as NET-inhibitor in the serum as previously shown (31). Plasma fibrinogen concentration in the VITT-patient was below the median of the control patients and stayed low until 7d (**Figures 6D–G**).

In summary, varying factors might contribute to the phenotype of distinct NET-formation in the VITT-patient as shown in histology (**Figure 2**): higher NET inducing factors, e.g. PF4 and antiPF4 (**Figure 3**), reduced NET-inhibitor fibrinogen (**Figures 6D–G**), as well as a higher amount of NET stabilization factors against endogenous nuclease like LL-37 (**Figure 5**).

### Cytokine Profiles in Blood of VITT-Patient *vs.* Controls

**Figure 7:**
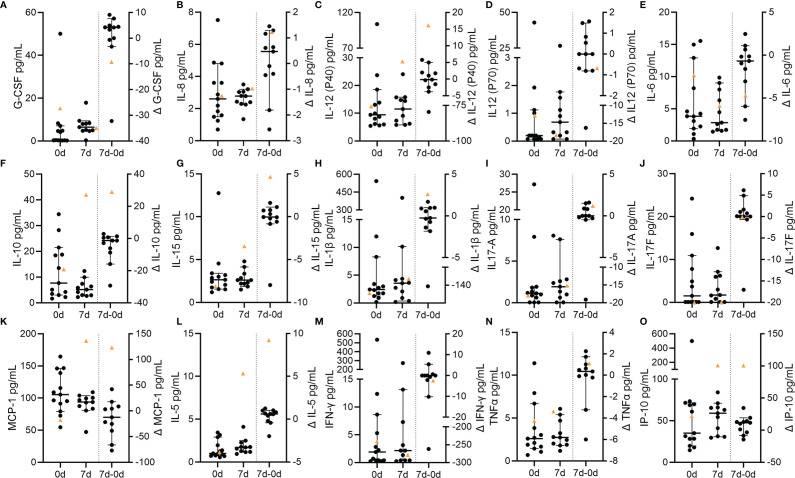
Circulating cytokines in VITT and control stroke patients. Concentrations of circulating cytokines at baseline and at follow-up as well as the temporal differences (Δ) between both time points. Individual data of each patient and the corresponding median and 95% confidence intervals of control values are depicted with data points of the VITT-patient highlighted as orange triangle. **(A)** C-CSF; **(B)** IL-8; **(C)** IL-12 (p40); **(D)** IL-12 (p70); **(E)** IL-6; **(F)** IL-10; **(G)** IL-15; **(H)** IL-1β; **(I)** IL-17A; **(J)** IL-17F; **(K)** MCP-1; **(L)** IL-5; **(M)** INF-γ; **(N)** TNF-α; **(O)** IP-10.

We finally analyzed cytokine profiles of the VITT patient and control patients and stratified the different cytokines in clusters associated to vaccination (IP-10, IL-6, IL-15) (21, 32), COVID-19 (G-CSF, IL-12, IL-8) (33–36), NET-formation (IL-6, IL-8, TNF-α, IL-17A) (37–39), LL-37 stimulation of immune cells (MCP-1, IL-8, IL-5, IL-1β, TNF-α) (39) and DNase activity (IL-6, IL1β, TNF-α, IFN-γ) (40, 41). Several interesting candidates showed exceptionally high delta values (0d-7d) in the VITT-patient (**Figures 7A–O**). A remarkable to strong increase in the VITT-patient was identified for IL-12 (P40), IL-1β, MCP-1, IL-5, IL-10, IL-15 and IP-10. A relative decrease in the VITT-patient was observed for G-CSF, IL-6 and IL12 (P70). Concentrations of IL-8, IFN-γ, IL-17A, and IL-17F in the VITT-patient did not substantially differ from the control group and did not change strongly over time. TNF-α was higher than the mean value of the control group at both time points and increased over time. In summary, the cytokine profile over time of the VITT-patient showed some differences compared to controls in eight out of 15 cytokines.

## Discussion

Beginning in March 2021, several cases of rare thrombotic complications after vaccination using ChAdOx1 have been reported worldwide with the majority being CVST but also ischemic strokes, thromboses of the splanchnic veins or pulmonary thromboses (1, 4, 42–44). It is unclear whether the relatively high incidence of CVST compared to other sites of thrombosis has a pathophysiological correlate or is an artifact, given that initially the majority of patients vaccinated with ChAdOx1 were younger and that CVST is a more common thrombotic disease in this age group. Indeed, the incidence of VITT associated CVST is highest in young female patients (44). In the meantime, arterial thrombosis due to VITT was reported in older patients, too. There is a tremendous need of individual predictors and therapeutic targets for thrombogenesis during VITT. Several hypotheses have been proposed for the pathophysiology of VITT. Since VITT is characterized by the presence of antibodies against PF4-polyanion complexes, similarities to the pathology of heparin induced thrombocytopenia (HIT) have been drawn in terms of an autoimmune variant of this disease. It was speculated that the vaccine-encoded severe acute respiratory syndrome coronavirus-2 (SARS-CoV-2) spike protein might be of relevance in contributing to the prothrombotic state (45), potentially explaining why SARS-CoV-2 infection itself also comes along with an enhanced risk for thrombotic complications. This hypothesis is further supported by the observation that thrombotic diseases were not evident after application of mRNA-based vaccines. However, while three motifs within the spike protein mimic immunogenic epitopes of PF4, antibodies against the SARS-CoV-2 spike protein did not cross-react with PF4 according to a study by Greinacher *et al (* 46). Thus, molecular mimicry based on a reaction towards the SARS-CoV-2 spike protein seems unlikely.

VITT was also reported after vaccination with another recombinant adenovirus vector-based vaccine, i.e. Ad26.COV2.S (Johnson&Johnson/Janssen) (47), leading to the hypothesis that adenovirus, or more precisely its DNA, might facilitate a prothrombotic state *via* interacting with PF4 (7, 48). Like Heparin, DNA has polyanion properties, and may be bound to PF4 and thereby create a neoantigen (49). Indeed, high amounts of PF4, antiPF4 and their combination can lead to compact NETs in high amounts in the absence of platelets (**Figure 3**).

COVID-19 increases the risk for thrombotic complications including ischemic stroke (50) and immunothrombotic mechanisms mediated by NETs have early been recognized as one important contributing factor (51). Interestingly, COVID-19 patients tested positive in PF4/heparin antigen tests without evidence of platelet-activating antibodies in functional tests (52).

In this study, we had the unique opportunity to investigate immunothrombotic mechanisms in thrombus and blood specimens obtained in the hyperacute setting of severe ischemic stroke due to VITT. In agreement with previous reports on thrombus analyses in CVST (53) and arterial thrombus specimens (7), we detected NET-specific markers, i.e. DNA-histone-1-complexes and MPO, in the immunohistological staining of the VITT-thrombus (**Figure 2** and **S2**). However, neither the intensity nor the pattern of NET-positive areas revealed remarkable differences compared to the control thrombi. NET markers were especially present in the outer shell of the thrombus and NETs proved to be degradable after *ex vivo* DNase treatment.

Interestingly, cfDNA and MPO blood levels at baseline were not distinct in the VITT case, while DNase activity was highest compared to controls (**Figure 4**). Concordantly, we found high amounts of DNase in the VITT-thrombus (**Figures 5E–G, I**). It is unclear, why these high levels of NET-degrading DNases should be insufficient *in vivo*. One explanation for this could be provided by the cathelicidin LL-37, which we were able to detect in high amount besides DNase in the VITT-thrombus (**Figures 5E–I**). Cathelicidins were previously found to be involved in platelet activation leading to arterial thrombosis (54). Interestingly, LL-37 was shown to modulate platelet reactivity, hemostasis, and thrombosis (55), which goes in line that in the index-patient at 7d high LL-37 levels were measured (**Figure 5D**) and further thrombotic events were observed at later time-points. Moreover, LL-37 was shown to protect NETs from degradation as it competes with DNase at the binding site of (28), implying a potential role in immunothrombosis. Therefore, the observed weak correlation of LL-37 and DNase inside the thrombus supports the hypothesis that LL-37 hinders the DNase degradation of NETs inside the thrombus, as DNase is not capable to degrade the DNA backbone where LL-37 is present (**Figures 5H, I**).

We also found a substantial elevation of LL-37 and of DNase activity in the index case over time, as well as an exclusive presence of C1q and C3d in the thrombus potentially indicating a regulatory response towards NETosis. Indeed, DNase and LL-37 values were higher in the VITT-patient compared to all controls (**Figures 4G, H** and **Figures 5A–D**). Values of cfDNA also showed a relative increase over time in the VITT-patient which however did not exceed that of all controls (**Figures 4A, B**). In this context, it also needs to be considered that cfDNA might originate from sources other than neutrophils (56).

Comparing the baseline with the follow-up cytokine values, we identified some biomarkers with clearly rising values over time in the VITT-patient (**Figure 7**). This particularly applies to pro-inflammatory cytokines and chemokines including IL-1β, MCP-1, IL-5 and CXCL10 (IP-10). IP-10 might be increased due to the vaccination itself as it is known to be associated with release of IFN-γ following vaccination. Of note, as described above, the VITT-patient suffered from a multitude of early adverse events and thus we are not able to provide evidence whether the temporal dynamics of biomarkers are cause or effect of these severe complications. However, there was also a relative decline of IL-6 (**Figure 7E**) values in the index patient making it unlikely that a general systemic inflammatory response leading to distorted biomarker values was caused by the adverse events alone. Interestingly, distinct pro-inflammatory cytokines were implicated in NETosis in the course of thrombotic diseases. This applies in particular to IL-1β which is crucial in inflammasome signaling that was shown to be of importance in NET-formation in cerebral thrombi (57). In myocardial infarction, NETs have been shown to induce MCP-1 secretion from endothelial cells and MCP-1 may in turn further aggravate NETosis (58). Of note, MCP-1 as well as IP-10 were previously implicated in thrombosis and were recently shown to be related to the clinical severity of COVID-19 (59). Considering the available data and the present study, the interplay between NETs and cytokines accordingly also appears to be important in the case presented here and for VITT in general.

Fibrinogen levels were low in the index patient, probably due to higher consumption during the excessive pro-thrombotic state (**Figures 6D–F**) and multiple subsequent thrombotic complications after the initial insult. In accordance with this hypothesis, fibrinogen levels were previously shown to be lower in patients with high clot burden and vice versa in acute stroke (60).

**Figure 8:**
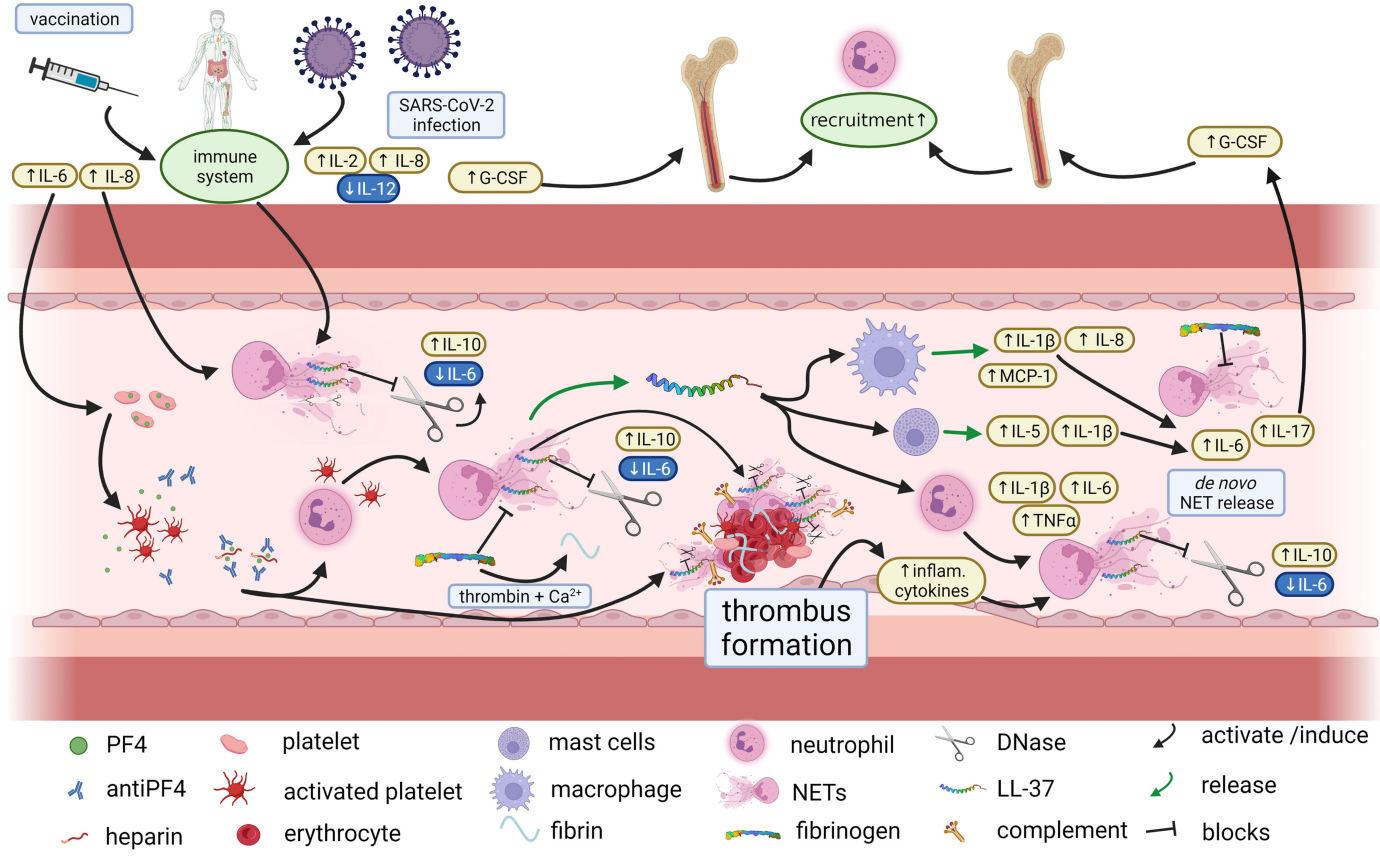
Proposed immunothrombotic mechanisms during VITT. Proposed interplay between cytokines and NETosis in VITT, Vaccination against SARS-CoV-2 may trigger the immune system and in particular NETosis as well as platelet activation by antiPF4 antibodies. NETosis is counter regulated by DNases which however may be blocked e.g. by LL-37. As a result, and under conversion of fibrinogen to fibrin excessive thrombus formation may occur with embedded NET markers, complement factors, LL-37 and DNase. The thrombus formation induces a subsequent inflammatory response which further contributes to NETosis in terms of a vicious cycle. Thus, an impaired efficacy of endogenous NET-regulation may be an important contributor to severe thrombotic complications of VITT (created with BioRender.com.).

Despite of the highest DNase activity, serum from the VITT-patient inhibited less efficiently ROS-induced NET-formation compared to controls *ex vivo* (**Figure 6**). This result may thus be a further indication for a disturbed NET-regulation in VITT. It was demonstrated that fetal calf serum contains heat stable nuclease that can degrade human NETs when it is used in cell culture systems (30). Furthermore, serum inhibits concentration dependent NET-formation of human neutrophils and serum concentration of 2% was described to be optimal for NET-induction analysis (61). Recently it was demonstrated that serum inhibits NET release of human neutrophils depending on the stimulus. Whereas NET release by lipopolysaccharides and calcium ionophores were inhibited by serum, PMA induced NETs in the presence of 2% serum after 3 hours incubation (62). Interestingly, Greinacher and colleagues identified that neutrophils isolated from healthy donors release NETs if they are incubated with serum from VITT patients and platelets from healthy donors (7). In our study we incubated the neutrophils with 12.5% serum and without the addition of platelets or any other stimuli and could not detect any NET-induction (**Figure S3B**), which indicates that no special NET inducer is inside of any tested serum sample, or the DNase activity is too high. It would be of interest to compare the NET-induction in future studies in presence of lower serum concentrations (2-5%) of the VITT-patient and the control serum samples in the absence of platelets. Nevertheless, as PMA induced NETs even in the presence of 12.5% serum (**Figure 6B**), it can be hypothesized that the phenotype would be even stronger with only 2% serum. Future studies could analyze the factor(s) that contribute to the PMA-induced NET-release in VITT serum. Based on the findings of this study and the existing literature, we hypothesize that NETs contribute to a vicious cycle of thrombogenesis during VITT (**Figure 8**). The fact that millions of SARS-CoV2 vaccinations have been administered in a short period of time has made it possible to detect the very rare cases of adverse events. In fact, such complications had not been observed in the according approval trials. The results of our work may provide an explanation at the level of innate immunity as to why a few patients are susceptible to thrombotic complications. It may be speculated that affected patients have reacted with an excessive thrombogenicity due to an underlying, previously unknown autoimmune susceptibility. Several studies indicated a crucial role of NETs in a variety of autoimmune diseases. Increasing circulating NETs in these conditions may result from reduced degradation rather than increased production (63). Emerging evidence suggests proteins from NETs as a source of autoantigens involved in pathogenesis of autoimmune diseases (64). Examples are: 1. anti-citrullinated peptides antibodies (ACPAs) in rheumatoid arthritis (RA) are directed against citrullinated histone 4 (65), 2. NET-associated anti-myeloperoxidase antibodies, 3. anti-protein 3 antibodies in anti-neutrophil cytoplasmic antibody (ANCA) associated vasculitis (AAV) and (66) 4. NET-derived extracellular nucleic acids and dsDNA are the targets of systemic lupus erythematosus (SLE) autoantibodies (67, 68). Interestingly, Hakkim and colleagues reported an impaired NET degradation in a subgroup of SLE patients based on either inhibition of DNase I or anti-NET antibodies, which prevent binding of DNase I to NETs. Both lead to a prolonged presentation of active NETs and their components accelerating immune responses and symptoms in these patients (69). Therefore, we hypothesize an impaired NET degradation in VITT patients, which might - similarly to SLE - aggravate auto-immunological mechanisms in general and moreover induce a vicious cascade *via* inflammatory pathways and complement activation to further NETosis and thrombembolic complications. The interplay between NETs and complement activation is also implicated in other endothelial injury syndromes: In pediatric patients with transplant-associated thrombotic microangiopathy (TA-TMA), a complication of hematopoietic stem cell transplantation (HSCT) associated with excessive complement activation, NETs were reported to directly link endothelial injury and complement activation leading to formation of microthrombi. After successful treatment of TA-TMA by complement inhibition dsDNA levels significantly declined. Gloude and colleagues suggest a IL-8 mediated mechanism of NET formation after endothelial injury (70). Of note, according to results of comprehensive immunological laboratory tests, no hints for known auto-immune disorders were found in the VITT-case.

Greinacher and colleagues indicated that anti-PF4 antibodies in VITT patients trigger NETosis (7). They detected markers of NETosis compared to healthy controls in VITT patient sera similar to our findings at day 7. In CVST, symptoms last in most cases over several days until it is diagnosed, whereas in patients with arterial thrombosis such as in stroke symptoms are fulminant and lead to immediate medical consultation. Therefore, it can be assumed that in our patient the baseline blood sampling was a lot earlier in clinical course of VITT than the samples in CVST and might explain the different findings. An impaired NETs degradation in this early stage is of even more importance and might raise the question on whether affected patients have reacted with an excessive thrombogenicity due to an underlying impairment of NETs degradation or whether the latter is consequence of the immune response. The antimicrobial peptide LL-37 is known to stabilize NETs against DNase degradation (28). We detected LL-37 in high amounts in the thrombus of our VITT patient in combination with DNase inside the thrombus and high DNase activity in blood. In contrast, in patients with MPO-ANCA-associated microscopic polyangiitis (MPA) DNase activity was shown to be reduced (71). It could be assumed that an impaired NET degradation triggering autoimmunity is induced by distinct mechanisms. Since antiPF4/heparin antibodies are rarely found in spontaneous CVST, it is likely that these are specifically involved in VITT (72).

Furthermore, it may be speculated whether an endogenous dysregulation of NETs also plays a role in other pathomechanisms with thrombotic complications, especially those for which no explanation is currently available. In this context, particular reference should be made to cryptogenic embolic strokes that account for up to 30% of all ischemic strokes. Meanwhile, it was repeatedly shown that cerebral thrombi contain NETs (73, 74) and it was speculated that mechanisms of immunothrombosis may be crucial in the pathophysiology of cryptogenic stroke. However, like in this work, the burden of NETs in thrombi of unclear origin did not substantially differ compared to clear stroke etiologies (75, 76).

Interestingly, an increased NETs-burden in thrombi as well as increased concentrations of distinct circulating cytokines were shown to be associated with a decreased likelihood of successful recanalization and resistance to thrombolysis (77–81). Of note, *ex vivo* administration of DNase was demonstrated to accelerate thrombolysis by tissue-plasminogen activator (79). We recently, however, did not find an association between endogenous DNase activity and reperfusion status in patients with acute stroke undergoing endovascular treatment (82). This may, in turn, indicate that individual endogenous mechanisms of NETs regulation exist that play an important role not only in the development of strokes of unclear causes but also in the probability of recanalization. More precisely, the actual efficacy of endogenous NETs degradation may differ inter-individually due to the aforementioned mechanisms with subsequent implications for the success of thrombus dissolution. This hypothesis merits further study.

There are several limitations of this work. Obviously, with one index case we can only provide descriptive and no inferential analyses. The conclusions deriving from our investigation thus need to be interpreted with caution and as hypothesis-generating. To allow for timely blood sampling in the hyper-acute setting of stroke, arterial blood was obtained from the access line at baseline instead of venous blood. Moreover, as discussed above, the VITT-patient suffered from a multitude of adverse events with potential effect on the biomarker values at follow up. Other control groups such as individuals after vaccination without VITT as well as patients with SARS-CoV-2-infection have not been considered and need to be addressed in future studies. The particular strength of this study is to have included a clinically comparable control collective as well as the combined analysis of blood and thrombus specimens in a multifaceted fashion.

## Conclusion

The results of this study suggest that not only an increased NETosis but also a disturbed endogenous degradation of NETs may be involved in thrombogenesis during VITT, contributing further to NETosis with a subsequent inflammatory response in the sense of a vicious cycle, and thus leading to an exaggerated pro-thrombotic state. Based on parallels to other auto-immune diseases, further insights into NETs-regulation are warranted to better understand the underlying pathophysiology of the pro-thrombotic coagulation state. This model could also be implicated in thrombembolic diseases of so far unclear cause, such as cryptogenic stroke.

## Data Availability Statement

The authors confirm that the data supporting the findings of this study are available within the published article. Raw data were generated at the Department of Neurology, Hannover Medical School, and the University of Veterinary Medicine Hannover, Department of Biochemistry. Derived data supporting the findings of this study are available from the corresponding author NdB (Nicole.de.buhr@tiho-hannover.de) and last author GMG (grosse.gerrit@mh-hannover.de) on request.

## Ethics Statement

The studies involving human participants were reviewed and approved by Ethics Committee of Hannover Medical School, Hannover, Germany. The patients/participants provided their written informed consent to participate in this study.

## Author Contributions

GG, RS, and NB wrote the first draft of the paper; GG, TB, JE, AL, MG, HW, KW, and RS contributed to the recruitment of patients and analyzed clinical data; AT and KW guided diagnosis and management of the index patient; CW, MK, and DJ performed histological analyses and interpretation; FG and PB contributed to interpretation of imaging data and sample acquisition; LF, RI, MM, RL, AT, MK-B, and NB performed laboratory analysis and interpretation of data; CF analyzed and interpreted cytokine data; All authors contributed to the article and approved the submitted version.

## Funding

This work was supported by the COVID-19 Research Network of the State of Lower Saxony (COFONI) with funding from the Ministry of Science and Culture of Lower Saxony, Germany (14-76403-184). GG was supported by PRACTIS—Clinician Scientist Program of Hannover Medical School, funded by the German Research Foundation (Grant number: DFG, ME 3696/3-1). LF was financially supported by the German Research Foundation (DFG) grants BU 3523/1-1, OH 166/2-1, and KO 3552/8-1. This Open Access publication was partially funded by the Deutsche Forschungsgemeinschaft (DFG, German Research Foundation) within the program LE 824/10-1 “Open Access Publication Costs” and University of Veterinary Medicine Hannover, Foundation.

## Conflict of Interest

The authors declare that the research was conducted in the absence of any commercial or financial relationships that could be construed as a potential conflict of interest.

## Publisher’s Note

All claims expressed in this article are solely those of the authors and do not necessarily represent those of their affiliated organizations, or those of the publisher, the editors and the reviewers. Any product that may be evaluated in this article, or claim that may be made by its manufacturer, is not guaranteed or endorsed by the publisher.
