# Supplementary material for: Insights Into Immunothrombotic Mechanisms in Acute Stroke due to Vaccine-Induced Immune Thrombotic Thrombocytopenia
Source: Front Immunol. 2022 May 10;13:879157. doi: 10.3389/fimmu.2022.879157 (PMC9128407; doi:10.3389/fimmu.2022.879157)
Supplement: Supplementary file 1 [file DataSheet_1.pdf]

Supplementary Material

1 Supplementary Figures and Tables

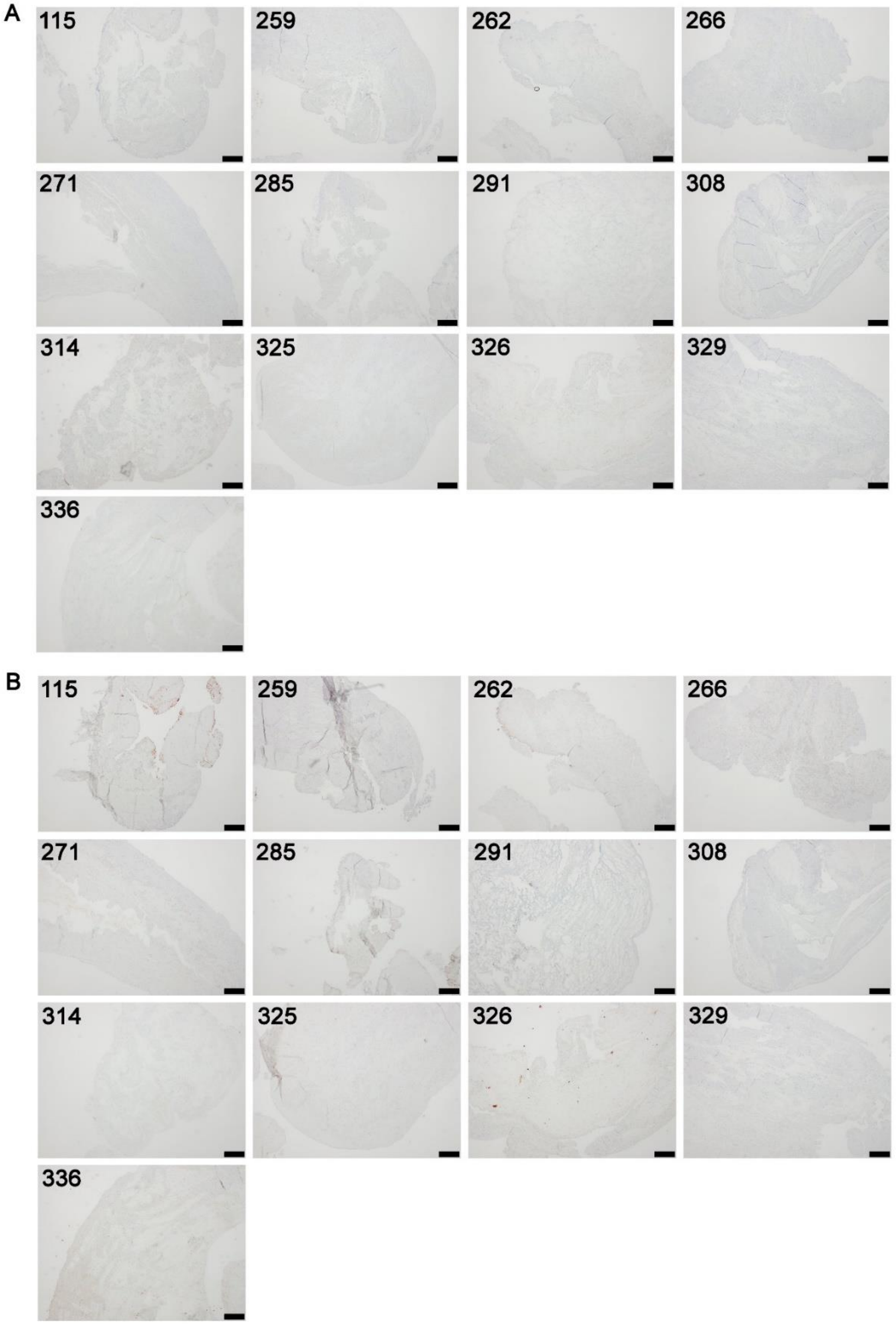

Supplemental Figure 1 C1q and C3d staining in control thrombi.

C1q (A) and C3d (B) staining as presented in Figure 1. Scale bar = 100µm

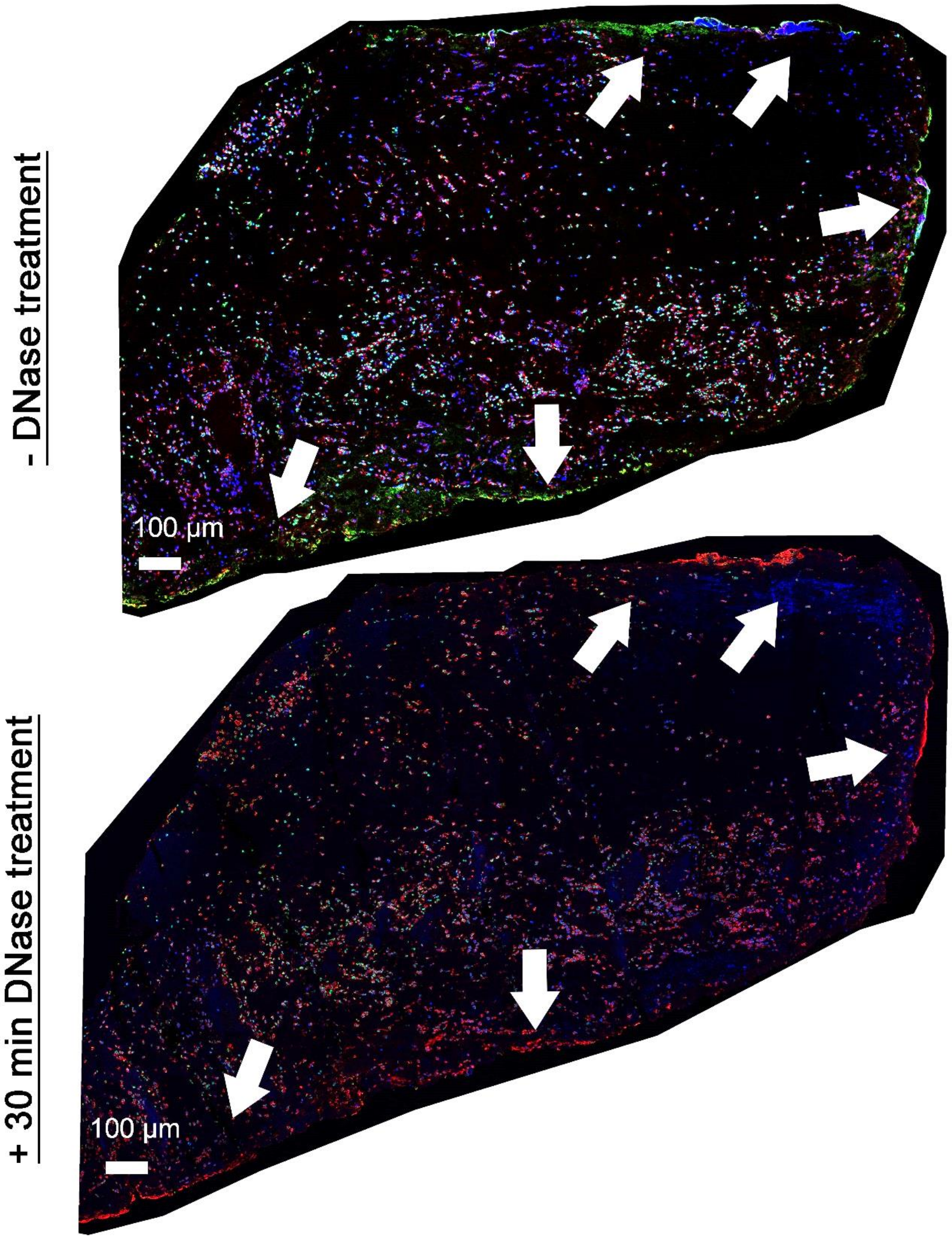

Supplemental Figure 2 Specific NET markers in thrombi of VITT patient

- Stitched images show distribution of NETs inside a further area of the thrombus (VITT patient) than presented in Figure 2 (blue = DNA, green = DNA-histone-1-complex, red = myeloperoxidase). White arrows depict strong NET signals (DNA-histone-1-complex and myeloperoxidase), especially in the outer range. Specificity of NET markers were identified after DNase degradation. Serial cuts of thrombi were treated during the NET-staining with and without DNase. DNase treatment deletes the DNA-histone-1-complex from thrombus section, but not the myeloperoxidase. The settings were adjusted to a respective isotype control (with and without DNase treatment).

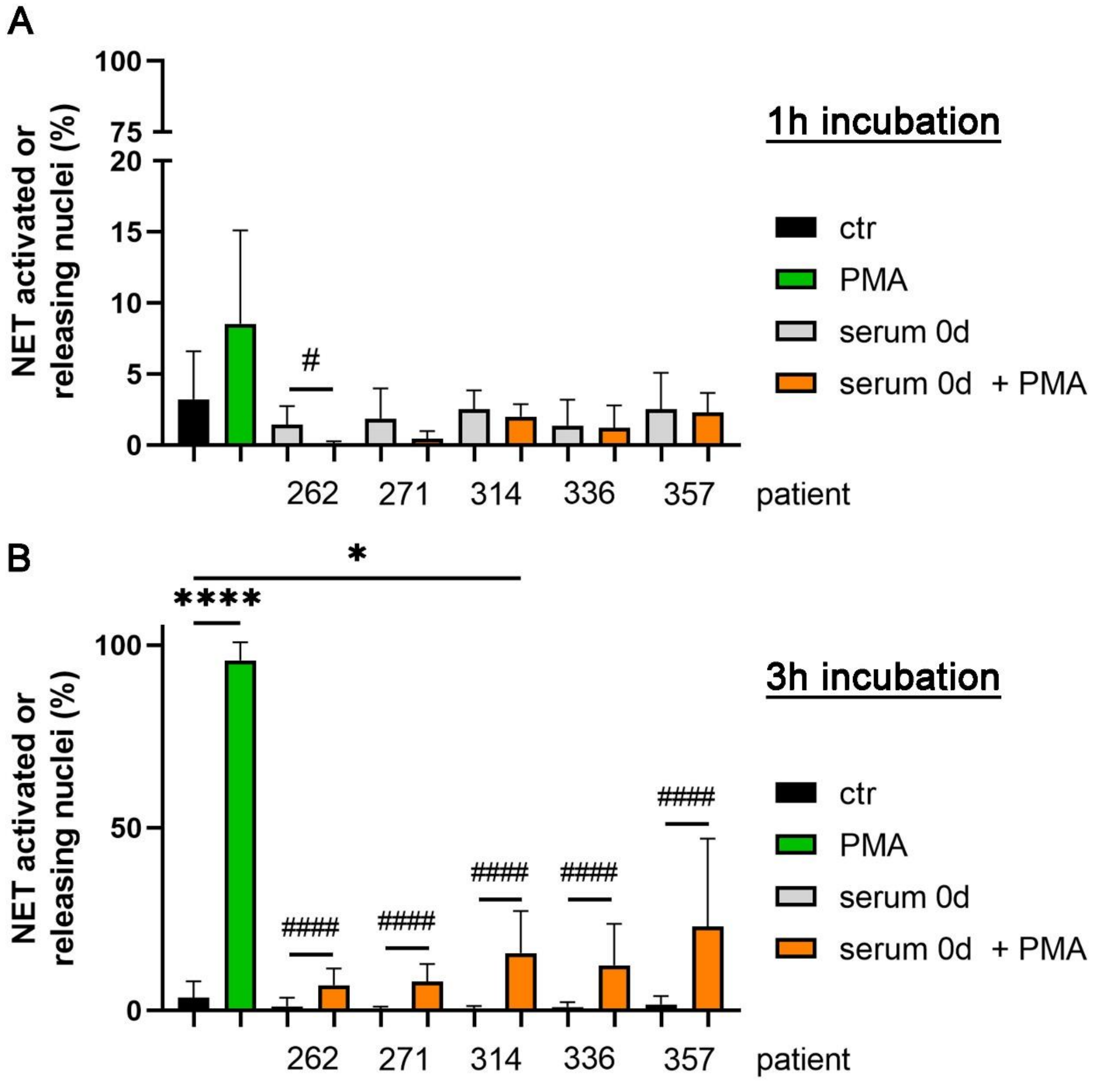

Supplemental Figure 3 Serum from VITT and control patients does not induce NET formation in absence of platelets and ROS-dependent NET release is reduced in presence of serum. Blood-derived neutrophils from healthy donors were incubated for 1 hour (A) and 3 hours (B) in serum of different patients. (A) After 1 hour the NET release starts only in the PMA control. (B) Whereas RPMI (ctr) did not induce NETs, PMA significantly induced NET formation after 3 hours. Neither serum from control collective patients (262, 271, 314 and 336), nor serum from the VITT patient (357) induced NETs. However, a ROS-dependent NET formation by PMA, was detectable, but was strongly decreased in presence of all serum samples. All data are shown as mean±SD of one technical run with duplicates for 1 hour and three technical runs with duplicates for 3 hours. In each sample three pictures were taken randomly. Therefore, data present the result out of 6 analyzed images (1 hour) and 18 analyzed images (3 hours). Statistical differences were analyzed with a one-way Kruskal-Wallis test ($p<0.0001$) followed by Dunn's multiple comparisons test. Significant differences are shown as follows: $^{*}p \leq 0.05$ and **** $p < 0.0001$. Furthermore, one-tailed Mann–Whitney test was used to analyze significant differences in each serum sample with and without PMA treatment. Significant differences are shown as follows: $^{\#}p \leq 0.05$ and $^{\#\#\#\#}p < 0.0001$.
